# Breaking the Cut‐Off Wavelength Limit of GaTe through Self‐Driven Oxygen Intercalation in Air

**DOI:** 10.1002/advs.202103429

**Published:** 2021-12-30

**Authors:** Renyan Zhang, Yuehua Wei, Yan Kang, Mingbo Pu, Xiong Li, Xiaoliang Ma, Mingfeng Xu, Xiangang Luo

**Affiliations:** ^1^ State Key Laboratory of Optical Technologies on Nano‐Fabrication and Micro‐Engineering Institute of Optics and Electronics Chinese Academy of Sciences Chengdu 610209 China; ^2^ Division of Frontier Science and Technology Institute of Optics and Electronics Chinese Academy of Sciences Chengdu 610209 China; ^3^ College of Advanced Interdisciplinary Studies National University of Defense Technology Changsha 410073 China; ^4^ Beijing Institute for Advanced Study National University of Defense Technology Changsha 410073 China

**Keywords:** bandgap shrinkage, gallium telluride, oxygen intercalation, photoconductivity, polarization‐sensitivity

## Abstract

Low symmetric two dimensional (2D) semiconductors are of great significance for their potential applications in polarization‐sensitive photodetection and quantum information devices. However, their real applications are limited by their photo‐detecting wavelength ranges, which are restricted by their fundamental optical bandgaps. Recently, intercalation has been demonstrated to be a powerful strategy to modulate the optical bandgaps of 2D semiconductors. Here, the authors report the self‐driven oxygen (O_2_) intercalation induced bandgap reduction from 1.75 to 1.19 eV in gallium telluride (GaTe) in air. This bandgap shrinkage provides the long‐wavelength detection threshold above ≈1100 nm for O_2_ intercalated GaTe (referred to as GaTe—O_2_), well beyond the cut‐off wavelength at ≈708 nm for pristine GaTe. The GaTe—O_2_ photodetectors have a high photoresponsivity, and highly anisotropic photodetection behavior to even sub‐waveband radiation. The dichroic ratio (*I*_max_ */I*_min_) of photocurrent is about 1.39 and 2.9 for 600 nm and 1100 nm, respectively. This findings demonstrates a broadband photodetector utilizing GaTe after breaking through its bandgap limitation by self‐driven O_2_ intercalation in air and further reveal its photoconductivity anisotropic nature. This provides design strategies of 2D materials‐based high‐performance broadband photodetectors for the exploration of polarized state information.

## Graphical abstract

This work reports a broadband photodetector utilizing GaTe after breaking through its bandgap limitation by self‐driven O_2_ intercalation in air and further reveals its anisotropic nature of the photoconductivity. This provides design strategies of 2D materials‐based high performance broadband photodetectors for the exploration of the polarized state information.

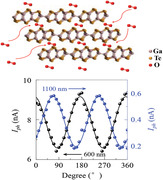

## 1 Introduction

The polarization‐sensitive photodetectors of the near‐infrared (750 to 1100 nm) spectral range are highly desirable for practical military and civil applications, such as spectroscopic analysis,^[^ ^1^ ^]^ optical communication,^[^ ^2^ ^]^ 3D sensing,^[^ ^2c^ ^]^ lidar sensor.^[^ ^3^ ^]^ Recently, the renaissance of low‐symmetric two dimensional (2D) semiconductors, which shows intrinsically in‐plane anisotropic optical and electronic properties,^[^ ^4^ ^]^ provides novel filter‐free freedom to harvest or manipulate the polarized‐state‐of‐light.^[^ ^5^ ^]^ Among them, black phosphorus (b‐P), with a bandgap of ≈0.3 eV in its thin‐film form (thickness >4 nm),^[^ ^6^ ^]^ could be used to detect near‐infrared radiation. However, applications for black phosphorus are limited by its drawbacks of ambient degradation.^[^ ^7^ ^]^ While, most other low‐symmetric 2D semiconductors, such as rhenium disulfide (ReS_2_) (Ref. [8]), gallium telluride (GaTe),^[^ ^9^ ^]^ are limited by their fundamental optical bandgaps, which are not suitable for the polarization‐sensitive near‐infrared photo‐sensing application.

The ability to break through the bandgap limitation is of great significance for practical optical and optoelectronic device applications.^[^ ^10^ ^]^ A few techniques have already been proposed to broaden the detection waveband of silicon via strain engineering,^[^ ^10a^ ^]^ the addition of dopants.^[^ ^11^ ^]^ While, for 2D semiconductors, the atomic thickness and dangling bonds free surface allow significant bandgap modifications through strain engineering,^[^ ^12^ ^]^ defects,^[^ ^10b^ ^]^ layer number control,^[^ ^13^ ^]^ alloying,^[^ ^14^ ^]^ as well as intercalation.^[^ ^15^ ^]^ Recently, short‐wavelength infrared photodetectors have already been reported using germanium (Ge)‐based chalcogenides, with the absorption cut‐off extended to the higher wavelength via intrinsic point defects.^[^ ^10b^ ^]^

Intercalation is a unique way to tune the physical properties, including the optical bandgap, of 2D materials through inserting guest species into the van der Waals gaps.^[^ ^15^, ^16^ ^]^ For example, the tin (Sn)^[^ ^17^ ^]^ and cobalt (Co)^[^ ^17^ ^]^ intercalation into the wide bandgap molybdenum trioxide (*α*‐MoO_3_) (about 2.8–3.6 eV)^[^ ^18^ ^]^ lead to a strong photo‐response in the visible to infrared broadband regions. Notably, it was found that O_2_ molecules will intercalate into the van der Waals gaps of GaTe in air spontaneously, accompanied by a significant bandgap reduction from ≈1.65 to ≈0.8 eV.^[^ ^15^, ^16^ ^]^ Therefore, the self‐driven O_2_ intercalation shall be of enormous interest for extending the photo‐detecting wavelength onset of GaTe toward the near‐infrared waveband.

In this work, we present a visible to near‐infrared broadband photodetector utilizing GaTe flakes in the air with a high photoresponsivity. This realization of near‐infrared photo‐response is attributed to the bandgap reduction of GaTe induced by the self‐driven O_2_ intercalation in air. The polarization‐sensitive properties of GaTe‐O_2_ photodetectors are also carefully investigated. The measured photocurrent peak/valley ratio is about 1.39 and 2.9 for 600 nm and 1100 nm, respectively. The combination of natural optical anisotropy and O_2_ intercalation extended detecting waveband endow GaTe competitive candidate for newly‐conceptual broadband (visible to near‐infrared) polarized‐light photodetector, and further motivate us to extend these materials toward the application of next‐generation photonic and optoelectronic devices.

## 2 Results and Discussion

### 2.1 Characterizations and Electrical Transport Properties of Multilayer GaTe Flakes in the Air

**Figure 1:**
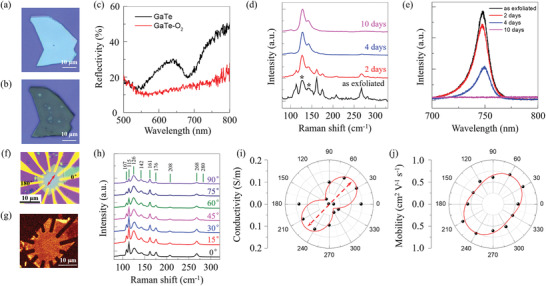
Optical and electrical transport properties characterizations of multilayer GaTe flakes in the air. a,b) Optical image of the GaTe flakes: as exfoliated and exposed to air for 10 days, respectively. c) The reflectivity spectra for GaTe before (black) and after exposure to air for 10 days (red), respectively. d,e) Raman and PL spectra for GaTe flakes after exposure to air for a different time: as‐exfoliated (black), 2 days (red), 4 days (blue), and 10 days (magenta), respectively. The Raman modes for GaTe—O_2_ are indicated by “*”. f) Optical image of a typical GaTe—O_2_ based FET with 12 electrodes spaced 30° apart. The *y*‐direction is labeled, and the scale bar is 10 µm. g) Corresponding Raman mapping of GaTe—O_2_ device with Raman modes of 115 cm^−1^. h) ARPRS of exfoliated GaTe flakes, with the sample rotated from 0° to 360° with the laser polarization direction. The excitation laser wavelength is 532 nm. The Raman modes are marked. Zero degrees is defined as the horizontal direction of the flakes in Figure S10, Supporting Information. g,h) Polar plot of angle‐dependent conductance and field‐effect mobility for GaTe‐O_2_ in (f), respectively.

**Figure 2:**
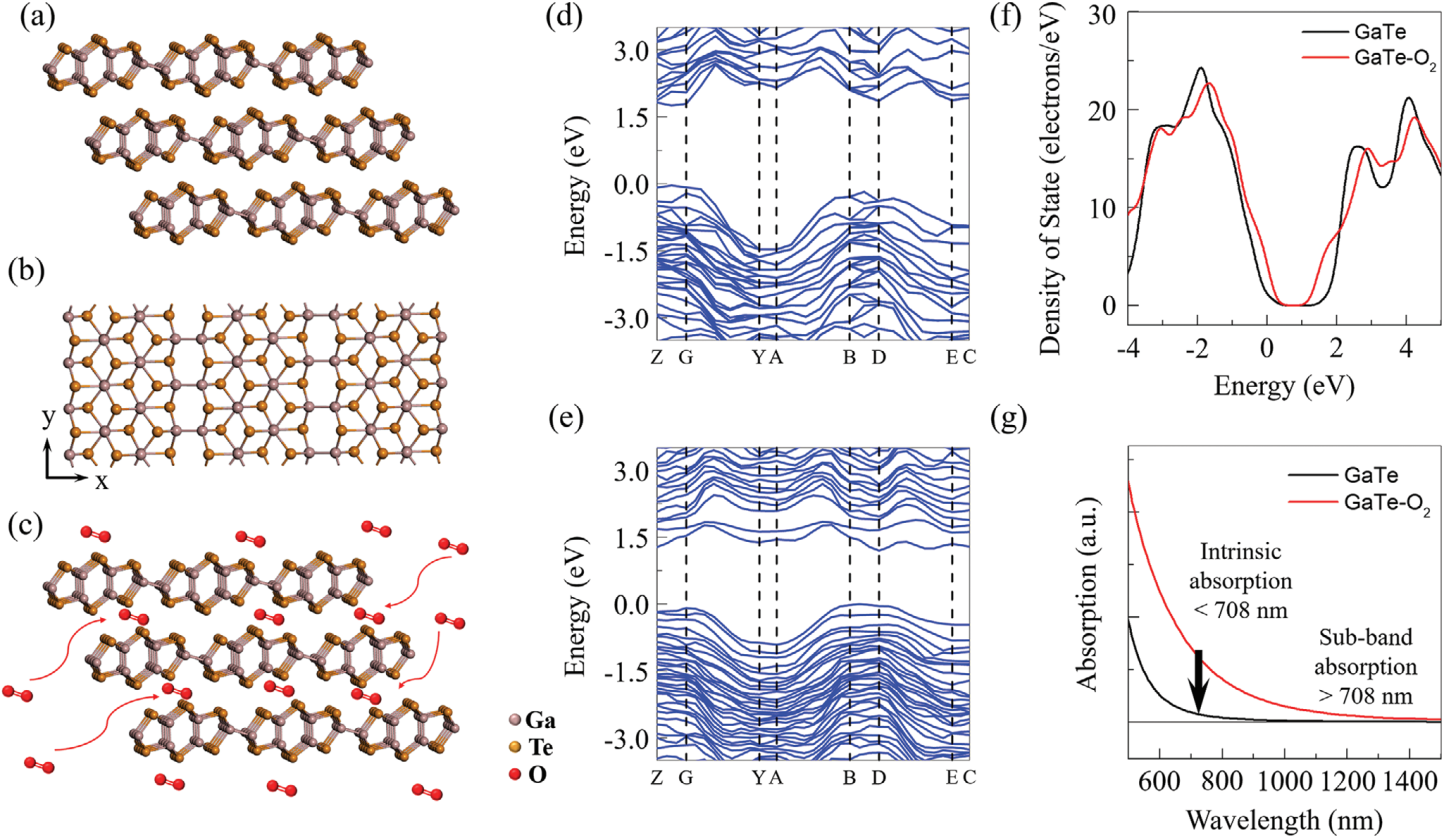
Bandgap reduction of GaTe by O_2_ intercalation. a,b) 3D view and top view of the crystal structures of GaTe, respectively. c) The cartoon schemes of O_2_ molecules intercalating into the van der Waals gaps of GaTe. d,e) Calculated electronic band structures of GaTe and GaTe—O_2_, respectively. f) The calculated DOSs of GaTe and GaTe—O_2_. g) Calculated optical absorption spectra of GaTe and GaTe—O_2_.

Ultra‐thin GaTe flakes with different thicknesses were mechanically exfoliated from commercial GaTe crystals (Nanjing Mukenano, China) onto silicon wafers with 300 nm silicon oxide. To demonstrate the air exposing change for GaTe flakes, optical and electrical characterizations were carried out (see Experimental Section for details). The optical images of the as exfoliated GaTe flake and the same flake after exposure to air for 10 days are shown in **Figure** **1**a,b, respectively. It shows that the GaTe flakes are clean and tidy without bubbles before and after exposure to air, unlike that in black phosphorus,^[^ ^19^ ^]^ where oxide island appears after air exposure. One may note that the white points in **Figure** **2**b, which are induced by the laser heating effect during the Raman and PL measurements (see Figure S1, Supporting Information, for comparison). The AFM measurements (Figure S2c,d, Supporting Information) show that the root means square (RMS) roughness of GaTe is only slightly increases from 0.9 nm for as exfoliated GaTe flake to 1.3 nm for 10 days air exposing sample. Simultaneously, the height of the GaTe flake (Figure S2e, Supporting Information) increases from ≈86.4 nm to ≈93.1 nm after air exposure, suggesting a small increase in interlamellar spacing.

More interestingly, as shown in Figure 1a,b, the color contrasts of GaTe flake change from light blue to grey–green after air exposure, indicating the change of optoelectronic properties. This is further confirmed by the optical reflectivity spectra in Figure 1c. For the typical as‐exfoliated GaTe flake on a silicon wafer with 300 nm silicon oxide, the reflectivity spectrum shows a valley at ≈682 nm, which can be attributed to the absorption edge of pristine GaTe.^[^ ^15b^ ^]^ After exposure to air, the reflectivity decreases for the wavelength range between 500 and 800 nm. This indicates that the optical absorption of GaTe increases after exposure to air, which means air exposing GaTe is a promising candidate for broadband photo‐detecting. The change of the optical properties cannot be explained by the surface oxidation, as the bandgap for tellurium dioxide (TeO_2_) or gallium(III) oxide (Ga_2_O_3_) is ≈3.8^[^ ^20^ ^]^ and ≈4.9 eV,^[^ ^21^ ^]^ respectively.

To analyze the chemical composition and stages of the GaTe compounds after air exposure, the energy‐dispersive X‐ray spectroscopy (EDS) and X‐ray photoelectron spectroscopy (XPS) measurements were carried out. The EDS spectra in Figure S3c, Supporting Information, yielded the average atomic percentages of O element for GaTe flakes before and after air exposing are 0.97% and 56.76%, respectively. Additionally, Figure S4, Supporting Information, shows that the O element distribution is fairly uniform in the air exposing samples. The XPS spectra for Ga 2p, Ga 3d, Te 3d, and O 1s are shown in Figure S5, Supporting Information. The doublet Te 3d peaks of 3d_5/2_ and 3d_3/2_ peaks for pristine GaTe is located at 572.8 and 583.2 eV, respectively. After air exposing, a new peak for Te 3d_5/2_ is found at 576.4 eV, indicating the Te‐O bonds formed during air exposing. Importantly, the newly emerging O 1s peak can be decomposed into a peak at 530.7 eV (Te—O bonds) and another peak at 532.7 eV (O—O bonds).^[^ ^22^ ^]^ Thus, although partial oxidation may be present, the Te‐O bonds cannot simply explain by the surface oxidation.^[^ ^15^, ^16^ ^]^ A considerable part of the O elements exist in O_2_ molecules and intercalate into the van der Waals of GaTe,^[^ ^15^, ^23^ ^]^ where the O_2_ chemisorbs to tellurium (Te—O bonds). The picture for structure change of GaTe after air exposure is consistent with that of the following density functional theory (DFT) calculation. For clarity, the air exposing GaTe (O_2_ intercalated GaTe) is referred to as GaTe‐O_2_ in the following part.

Raman spectra (unpolarized) for the as exfoliated GaTe flakes with different thicknesses are shown in Figure S6, Supporting Information, where the Raman vibration modes exhibit an obvious thickness‐dependent behavior. Nine Raman modes at 107, 115, 126, 142, 161, 176, 208, 268, and 280 cm^−1^ are clearly resolved for as exfoliated thicker GaTe flakes (>30 nm), consistent with previous researches.^[^ ^24^ ^]^ The two broad Raman modes at 126, 142 cm^−1^ are attributed to the GaTe—O_2_,^[^ ^15b^ ^]^ with the full‐wide at half maximum (FWHM) of 10.85 and 9.79 cm^−1^ for the 88 nm GaTe flake, respectively. Figure 1d shows the evolution of the Raman spectra for ultrathin GaTe flakes after being exposed to air for various times. The intensity for Raman modes at 126, 142 cm^−1^ is increasing and then become dominant with the increase of the air exposing time, indicating that the amount of O_2_ increases with the increase of air exposing time. Notably, the Raman analysis indicates that O_2_ intercalation happens spontaneously during this complicated preparing process in air, similar with that in the self‐intercalation process in some 2D materials,^[^ ^25^ ^]^ different from that in their bulk counterpart, which needs a couple of days for the O_2_ concentrates in the bulk.^[^ ^15^, ^26^ ^]^ In addition, only two broad Raman vibration modes at 126 and 142 cm^−1^ are obvious for as exfoliated thinner GaTe flakes (≈11 nm), which indicates that this few‐layer GaTe flake is fully transformed during the exfoliation process.

Besides the Raman spectra, we have also studied the evolution of photoluminescence (PL) of GaTe flakes with air exposing time (Figure 1e). As exfoliated GaTe shows a PL peak at 747 nm with the excitation laser of 532 nm and the intensity of PL peak is increase with the thickness of GaTe nanosheets (Figure S7, Supporting Information), consistent with previous researches.^[^ ^9^, ^27^ ^]^ In Figure 1e, the PL intensity decrease with the air exposing time. After 10 days of air exposure, the PL peak is completely disappeared. The loss of PL signal indicates a direct to indirect band structure from GaTe to GaTe—O_2_.

To further analyze the effect of the atmosphere on the aforementioned changes, the evolution of multilayer GaTe flakes exposure to different atmospheres were analyzed, including humidified O_2_, dry air, and humidified N_2_. Figure S8, Supporting Information, shows that the Raman and PL spectra remain almost unchanged as that of as exfoliated samples after 10 days of exposure to dry air and humidified N_2_. While, humidified O_2_ exposure induces a higher rate of change, where the PL signal completely disappears in 1 day. Above all, O_2_ and moisture are necessary for the aforementioned changes. In addition, the air exposing change is partially reversible by annealing in an Ar atmosphere at the temperature of 350 ℃. Figure S9a, Supporting Information, shows that the 126 and 142 cm^−1^ Raman modes disappear after 30 min of annealing for as exfoliated GaTe, and the corresponding PL intensity increase in Figure S9b, Supporting Information. However, the Raman spectra for the full transferred GaTe—O_2_ sample remain almost unchanged for a rather long time anneal in Ar. Although, the PL signal partially reappears.

Previous researches show that the scattered Raman vibration intensity reaches the maximum with excitation laser (wavelength of 532 nm) polarization along the y crystalline direction for the Raman modes at 107, 115, 126, 176, 208, 268 cm^−1^ and minimum for 280 cm^−1^ Raman mode.^[^ ^24^ ^]^ The angle‐resolved polarized Raman spectroscopy (ARPRS) characterization was carried out by revolving the samples in the x–y plane under the excitation of a linearly polarized incident laser of 532 nm. Figure 1h presents a series of ARPRS spectra for the as exfoliated GaTe flake in Figure S10, Supporting Information, with a thickness of ≈88 nm, with different angles *θ* between the light polarization and the horizontal direction. The polarized Raman intensities have a strong correlation with laser polarization angle *θ*. The polar plots of the ARPRS intensity are summarized in Figure S11, Supporting Information for the Raman modes of 107, 115, 126, 142, 161, 176, 208, 268, and 280 cm^−1^. The Raman modes of 107, 115, 126, 176, 208, 268 cm^−1^ show a maximum at an angle of about 8° and show a period of 180°. And the Raman mode 280 cm^−1^ shows a maximum at an angle of about 98° with twofold symmetry. Thus, the crystalline orientation of the exfoliated GaTe nanosheet can be determined as that the y crystal orientation is identified along the 8°‐188° direction, as indicated by the red line in Figure S10, Supporting Information. For clarity, the polar plot of Raman mode of 520 cm^−1^ of silicon is also given in Figure S12, Supporting Information, which is isotropic, that is, the substrate does not affect the anisotropic response of GaTe flakes. In addition, Figures S13 and S14, Supporting Information, show ARPRS spectra of two other typical as exfoliated GaTe flakes with the thickness of 320 and 11 nm, respectively, which shows a similar twofold symmetry as in the 88 nm sample. Notably, the 126 cm^−1^ Raman modes for GaTe—O_2_ remain anisotropic, which indicates that monoclinic GaTe is still in its initial symmetry of phases after air exposure and remains anisotropic.^[^ ^31^ ^]^

Besides the Raman anisotropy, we have also studied the angle‐resolved PL of as exfoliated GaTe flakes. The angle‐resolved PL intensity shows angular dependence with the incident light polarization angles, having a two‐fold symmetry (Figure S15, Supporting Information). The maximum PL intensity at the y chain direction is about two times larger than at the x chain direction, indicating strong anisotropy of the optical response of the GaTe flakes on the silicon wafer. This demonstrates a high optical in‐plane anisotropy, indicating that GaTe is a promising candidate for the applications of linear‐polarization‐sensitive photodetectors.

The anisotropic electrical transport properties of GaTe—O_2_ flakes were probed by angle‐resolved electrical transport measurements from a 12‐terminal back‐gated GaTe—O_2_ field‐effect transistor (FET) at room temperature in air. Figure 1f shows that the Cr/Au (5/50 nm) metal contacts were deposited on GaTe‐O_2_ flake (the thickness is about 100 nm), spaced at angles of 30° between every two adjacent electrodes, and the six pairs of opposite electrodes have the same channel length (≈11 µm, wide ≈2 µm). The corresponding Raman mapping of GaTe—O_2_ device with Raman modes of 115 cm^−1^ is given in Figure 1g, where the area of GaTe—O_2_ is clearly shown. The 0° and 180° electrodes are labeled in Figure 2f. The y crystalline direction is determined along 45–225° direction by ARPRS. The *I*_ds_ (drain–source current) − *V*_ds_ (drain–source bias voltage) testing is performed along with each pair of diagonal electrodes for a transistor geometry. The direction‐dependent *I*_ds_–*V*_ds_ curves are shown in Figure S16a, Supporting Information, which are linear, indicating a good ohmic contact property between GaTe—O_2_ flakes and Cr/Au electrodes. The *I*_ds_–*V*_ds_ curves have clear angular dependence. The angle‐dependent electrical conductance of these two‐terminal devices is shown as a polar coordinate in Figure 1i, where maximum conductivity is 0.151 S m^−1^ along the y‐direction, and minimum conductivity is 0.028 S m^−1^ along the x‐direction. Figure S16b, Supporting Information, presents the corresponding transfer characteristics of the GaTe–O_2_ transistor with different angles when the back gate voltage varies from −50 to 50 V. The *I*_ds_ of GaTe–O_2_ transistors increase with lower negative *V*_g_, revealing an obvious p‐type semiconducting behavior with the on/off ratio of about 200, which is consistent with previous reports.^[^ ^9^, ^15^, ^28^ ^]^ The obtained field‐effect mobility is plotted as a function of direction in Figure 1j, which has a twofold ellipsoidal oscillation. The largest mobility of 0.77 cm^2^·V^−1^·s^−1^ occurs in the 45° (225°) direction, while the minimum of 0.51 cm^2^·V^−1^·s^−1^ is at 135° (315°). Thus, the y‐direction conductivity/mobility is 5.42/1.52 times larger than that along the x‐direction for GaTe–O_2_ flakes. This is comparable with that of other reported anisotropic 2D crystals, such as b‐P (≈2.5).^[^ ^6^ ^]^

In addition, we further measure the photo‐response properties of the 12‐terminal back‐gated GaTe‐O_2_ FETs in Figure 1f at room temperature in air. The *I*_ds_–*V*_ds_ curves along 0°–180° direction with and without laser irradiation are shown in Figure S17, Supporting Information, where one can see a clear increase of the current under the irradiation of 532 and 633 nm laser with the power density of 50 mW cm^−2^. The current–time curves under 532 and 633 nm irradiation with different laser power densities from 10 to 50 mW cm^−2^ are shown in Figure S18a,b, Supporting Information, respectively. And, the power density‐dependent photocurrent (*I*_PC_) and photoresponsivity (*R*) are summarized in Figure S18c, Supporting Information. Besides, the photo‐response along different directions is also measured, with the polar plot of the photo‐responsivity as a function of direction shown in Figure S20c, Supporting Information. This anisotropy of the photo‐response (Figure S20, Supporting Information) of 532 and 633 nm is attributed to the anisotropic transport properties of GaTe—O_2_ flakes.

### 2.2 Bandgap Restructuring of GaTe Induced by O_2_ Intercalation in Air

To further understand the effect of O_2_ intercalation, we carried out DFT calculations of the band structure of GaTe and GaTe—O_2_. The GaTe flakes in this work are above 50 nm, which shows the bulk properties. Thus, the DFT calculation for the bulk crystal of GaTe is carried out before and after O_2_ intercalation (see Experimental Section for details). GaTe, a layered III‐VI semiconductor, has a monoclinic crystal structure of the C_2h_^3^ space‐group^[^ ^5^, ^9^, ^24^, ^29^ ^]^ (Figure 2a,b), with the unit cell and first Brillouin zone shown in Figure S21, Supporting Information. O_2_ molecules will intercalate into the van der Waals gaps of GaTe in the air (Figure 2c).^[^ ^15^, ^16^, ^26^ ^]^ For O_2_ intercalated GaTe (referred to as GaTe—O_2_), one O_2_ molecule is added into the van der Waals gaps of the unit cell of GaTe,^[^ ^15^, ^16^, ^26^ ^]^ with O_2_ molecules chemisorbs to Te atoms, as shown in Figure S21, Supporting Information. The DFT calculated band structure for GaTe and GaTe–O_2_ are shown in Figure 2d,e, respectively, which shows that the self‐driven O_2_ intercalation induces a bandgap reduction from 1.75 to 1.19 eV for GaTe flakes in the air. In addition, a direct to indirect band structure transition happens with O_2_ intercalation, consistent with the PL measurements in Figure 1e. Additionally, we found that this bandgap reduction maintains true for the oxygen‐chemisorbed monolayer GaTe in the air (Figure S22, Supporting Information).

Figure 2f shows the corresponding density of state (DOS) for GaTe and GaTe—O_2_, where low‐lying sub‐bands associated with the new conduction band form in GaTe—O_2_. These new sub‐bands, composed of Te‐p, O‐p, Ga‐s, and Ga‐p states (Figure S23, Supporting Information), are expected to induce distinct optical properties. Calculated optical absorption spectra GaTe before and after O_2_ intercalation is shown in Figure 2g, where the extrinsic absorption beyond the cut‐off wavelength of GaTe at ≈708 nm appears in GaTe—O_2_. Thus, the detection waveband for GaTe—O_2_ is expected to be extended to the near‐infrared wavelength. However, the extrinsic sub‐band absorption of GaTe—O_2_ is much smaller than the intrinsic absorption, due to smaller DOS for the sub‐bands.

### 2.3 Photo‐Response of GaTe Before and After Air Exposure

**Figure 3:**
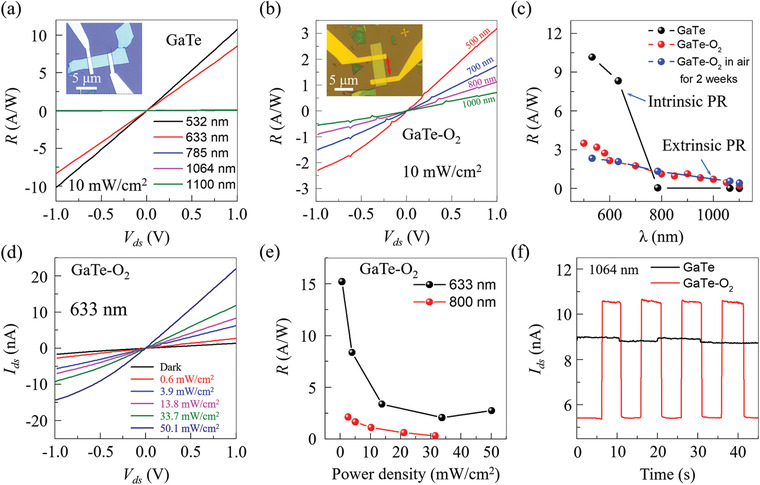
Photo‐response properties of multilayer GaTe photodetector before and after exposure to air. (a, b) *R–V*_ds_ curves of GaTe and GaTe—O_2_ FET under laser radiation (10 mW cm^−2^) with different wavelengths, respectively. Inset: optical images of GaTe and GaTe‐O_2_ FET, with the scale bar of 5 µm. The y crystal direction is indicated by the red line in the inset of (b). c) Photoresponsivity of GaTe and GaTe—O_2_ device as a function of wavelength. PR is the abbreviation for photo‐response. d) *I*_ds_–*V*_ds_ curves of GaTe—O_2_ device under laser radiation of 633 nm under different laser power densities. e) Photoresponsivity of GaTe—O_2_ device as a function of laser power density. f) The dependence of *I*_ds_ with times with irradiation laser wavelength of 1064 nm for GaTe (black) and GaTe—O_2_ (red).

The optoelectronic properties of pristine GaTe and GaTe—O_2_ flakes were further probed by exploring the two‐terminal back‐gated FETs. The GaTe FET is fabricated using the shadow mask method and annealing in Ar atmosphere (Figure S24, Supporting Information), and GaTe—O_2_ FET is fabricated through an optical lithography process in the air (see Experimental Section for details). The GaTe and GaTe—O_2_ devices measure in vacuum and air, respectively. During photo‐response measurements, monochromatic light illumination was directed vertically onto functional channels. The *R* as a function of *V*_ds_ with the incident light intensity of 10 mW cm^−2^ at different radiation laser wavelengths for GaTe and GaTe—O_2_ devices are shown in **Figure** **3**a,b, respectively. The linearity of *R*–*V*_ds_ curves indicates the ohmic contact between the electrodes and GaTe and GaTe—O_2_ nanosheet. The *R* at *V*_ds_ = 1 V as a function of wavelength from 500 to 1100 nm was summarized in Figure 3c. The *R* at 10 mW cm^−2^ increases with decreasing wavelength, which can reach 10.2 and 3.3 A W^−1^ with the wavelength of 500 nm for GaTe and GaTe—O_2_ flakes, respectively. The *R* for pristine GaTe device is much higher than that for GaTe—O_2_ device, which is attributed to the direct to indirect bandgap transition. More importantly, we found that the GaTe—O_2_ photodetectors show an obvious long decay tail from the visible to the near‐infrared detection (Figure 3b,c, and Figure S25, Supporting Information), that is, the detection wavelength of GaTe photodetector is broadened to above 1100 nm, much longer than that of the detection cut‐off wavelength of pristine GaTe at <708 nm. The extended photo‐response range cannot be simply explained by the surface oxidation, considering the large bandgap for TeO_2_ (≈3.8 eV^[^ ^20^ ^]^) and Ga_2_O_3_ (≈4.9 eV^[^ ^21^ ^]^). Thus, this sub‐waveband photo‐response is attributed to the bandgap reduction by O_2_ intercalation. It needs to mention that the responsivity *R* for intrinsic photo‐response is much larger than that of the extrinsic photo‐response, due to the higher DOS in valance and conductive band than the sub‐bands, agreed quite well with DFT calculation in Figure 2.

Figure 3d presents the *I*_ds_–*V*_ds_ curves for GaTe‐O_2_ device under irradiation of 633 nm with power density ranging from 0 to 50.1 mW cm^−2^. The incident light power density dependence of *R* for 633 and 800 nm is given in Figure 3e, which shows that *R* decreases with the increase of power density. And R is about 15 A W^−1^ for 633 nm at a power density of 0.6 mW cm^−2^. Figure 3f shows the time‐resolved responses of GaTe and GaTe—O_2_ phototransistors at a bias voltage of 0.5 V for illuminate light (power density of 10 mW cm^−2^, wavelength of 1064 nm) on and off. Compared to GaTe, the GaTe—O_2_ phototransistors shows a clear photo‐response for the wavelength of 1064 nm, consistent with the above analysis. To further uncover the intrinsic mechanism, we investigated the time‐resolved photo‐response of the GaTe—O_2_ device. The response speed of the GaTe—O_2_ photoconductor at 500, 750, and 1000 nm is investigated, which reflects the capability of following a varying optical signal (Figure S25, Supporting Information). Figure S26, Supporting Information, shows the time‐resolved responses of GaTe—O_2_ phototransistors at a bias voltage of 0.5 V for illuminate light (633 nm) with different power densities from 0.4 to 50.1 mW cm^−2^. The response time is estimated as ≈0.1 s, as shown in Figure S27, Supporting Information. The slow response is probably limited by indirect band structure, the high contact resistance, and the defect caused by O_2_ intercalation. The constructed GaTe—O_2_‐based phototransistor exhibits excellent stability during the measurement. After exposure to air for two weeks, no change is observed for the responsibility for GaTe—O_2_ phototransistors in Figure 3c and Figure S28, Supporting Information.

### 2.4 Polarization‐Sensitive Photo‐Response of GaTe‐O_2_ Photodetector

**Figure 4:**
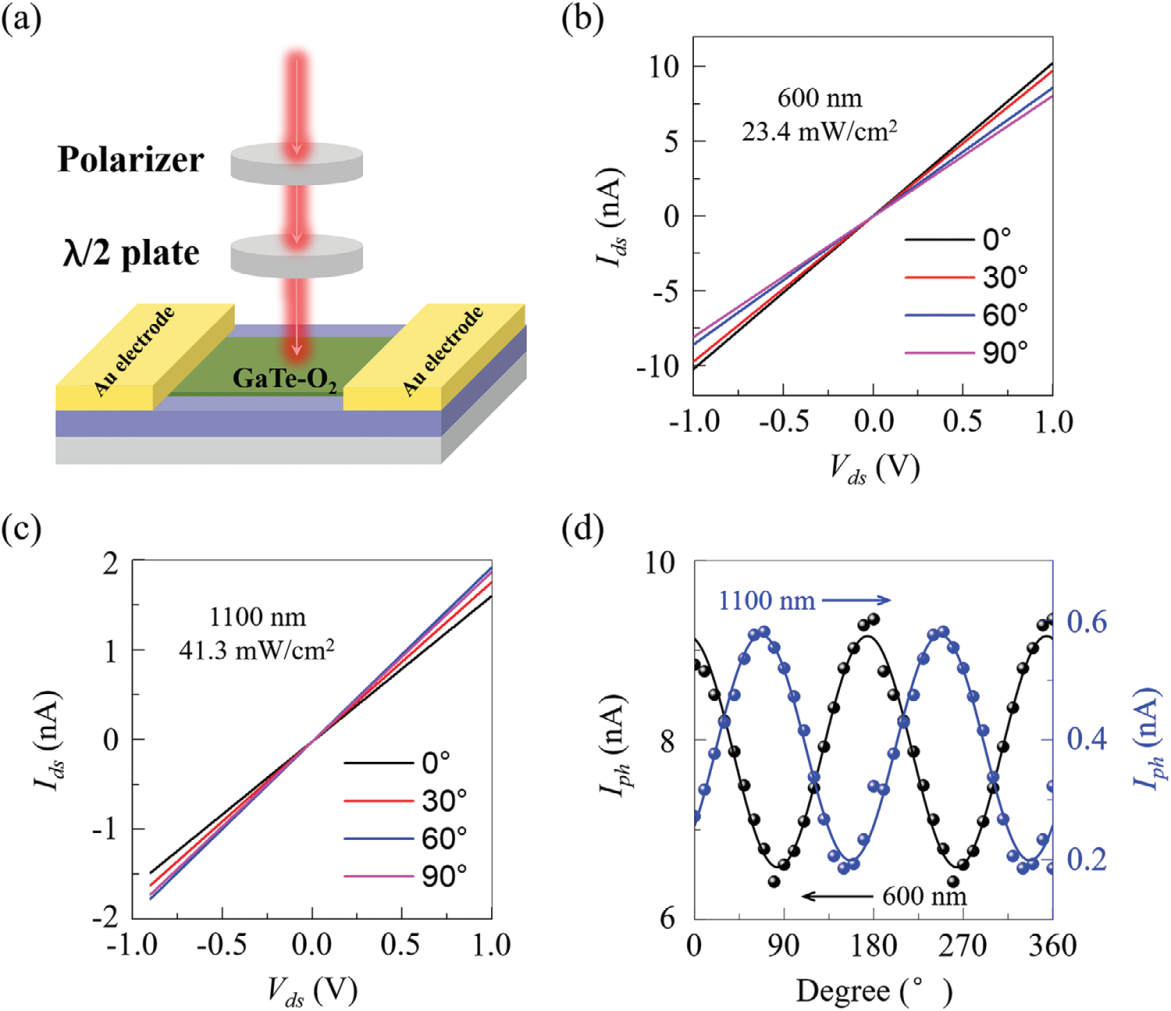
Polarization‐dependent photocurrent of multilayer GaTe‐O_2_ photodetectors. a) Schematic polarization‐dependent photocurrent measurement system. b) Direction‐dependent *I*_ds_ *–V*_ds_ curves under 600 nm laser radiation with the power density of 23.4 mW cm^−2^. c) Direction‐dependent *I*_ds_ *–V*_ds_ curves under 1100 nm laser radiation with the power density of 41.3 mW cm^−2^. d) Angle‐resolved photocurrent of GaTe‐O_2_ photodetector under 600 and 1100 nm laser radiation.

To explore the anisotropic photo‐response nature of GaTe‐O_2_ FETs, we performed angle‐resolved polarization‐dependent photocurrent measurements on the GaTe—O_2_ FET (the inset of Figure 3b) at room temperature in air. As illustrated in **Figure** **4**a, the laser passes through a polarizer and a half‐wave plate and exposures on GaTe‐O_2_ photodetector. Figure 4b,c show a series of *I*_ds_–*V*_ds_ curves with different laser polarizations angle recorded with exciting laser wavelength of 600 nm power density of 23.4 mW cm^−2^ and 1100 nm 41.3 mW cm^−2^, respectively. The polarization angle 0 is indicated in the device optical image in the inset of Figure 3b. Figure 4d shows the dependence of photocurrent on the polarization angle for the wavelength of 600 nm and 1100 nm. For a wavelength of 600 nm, the photocurrent demonstrated maximum photo‐response at *θ* = 0°, 180°, and 360° with the light polarizing parallel to the *y*‐direction of GaTe, while the minimum photocurrent is observed at *θ* = 90° and 270° with the light polarization being along x crystal direction of GaTe. It should be emphasized that the GaTe—O_2_ detector exhibits highly‐anisotropic photo‐responses to sub‐waveband polarized‐light of 1100 nm, with the maximum signal intensity observed at an angle around 60° and 240°, and minimum intensity at an angle around 150° and 330°. Repeating this process, that is, the successive rotation of polarized‐light angle from 0° to 360°, the dichroism results of photocurrent maximum and minimum can be easily repeated. The “W” shape curve is also observed for 1100 nm under different power densities in Figure S29, Supporting Information, which can be fitted by the sinusoidal function. The photocurrent dichroic ratio (peak to valley ratio) for the wavelengths of 600 and 1100 nm is about 1.39 and 2.9, respectively, indicating strong polarization‐sensitive photodetection. The obtained dichroic ratio here is compatible with previous polarization‐sensitive photodetectors.^[^ ^10b^ ^]^ The angle‐dependence of the extrinsic photoconductivity is slightly sample‐dependent, which is attributed to the O_2_ intercalation induced disorder and grain reorientation.^[^ ^16a^ ^]^ The most likely reason for the anisotropy in the photocurrent is anisotropic optical absorption due to the anisotropy on the refractive index.^[^ ^26^, ^30^ ^]^ The polarization‐sensitive photo‐response is also measured for the wavelength of 500 and 800 nm (Figure S30, Supporting Information), which also shows a strong polarization sensitivity. Here, we can see that the photo‐response of GaTe—O_2_ is anisotropic. The anisotropic photo‐response properties of air‐exposed GaTe agree with the 126 cm^−1^ Raman modes anisotropy in Figures S11–S14, Supporting Information. This is also consistent with previous research^[^ ^31^ ^]^ that monoclinic GaTe maintains its initial symmetry of phases after air exposure and remains anisotropic.

## 3 Conclusions

In summary, we demonstrate that the detecting wavelength of GaTe photodetector in the air is above 1100 nm, much longer than the cut‐off wavelength of pristine GaTe at ≈708 nm. The extension of the detecting wavelength range is attributed to the bandgap reduction from 1.75 to 1.19 eV by the self‐driven O_2_ intercalation in air, which is carefully analyzed by DFT calculation. The photo‐response of GaTe—O_2_ device is systematically studied, which has a high photoresponsivity and short response time. In addition, the polarization‐sensitive properties of GaTe—O_2_ photodetectors are characterized by the angle‐resolved polarization‐dependent photocurrent measurements. The GaTe—O_2_‐based phototransistor shows highly anisotropic photodetection behavior to even sub‐waveband radiation, with a peak/valley ratio of 1.39 and 2.9 for 600 and 1100 nm, respectively. Our finding provides new insight into the extrinsic photoconductivity and anisotropic physical properties of 2D materials, which is of great significance for both fundamental researches and real applications.

## 4 Experimental Section

### Materials Preparation and Characterizations

All the GaTe flakes preparation and characterizations were carried out in air at room temperature (≈25 ℃) with the relative humidity ≈70%, unless otherwise stated. Multilayer GaTe flakes were prepared by mechanically exfoliation method from bulk GaTe crystals (Nanjing Mukenano, China) onto the silicon wafer with 300 nm oxides. The LV150N optical microscopy (Nikon, Japan) was adopted to identify these GaTe flakes. The thickness and RMS roughness of these flakes were determined by atomic force microscopy (AFM) (NTEGRA, NT‐MDT, Russia). The EDS analyses were carried out by Oxford Instruments X‐Max detector integrated with the Thermo Scientific Apreo 2C scanning electron microscope (SEM) (Thermo Scientific, USA).

The Nexsa X spectrometer (Thermo Scientific, USA) was used to obtain XPS spectra. The sample size for XPS analysis is typical ≈2 mm × 2 mm. The fresh GaTe surface was obtained by mechanical exfoliation using Scotch tape. While GaTe–O_2_ surface was obtained by mechanical exfoliation and then exposed to air for 10 days. During XPS measurements, the signal was taken from the area with the size of 400 µm in diameter, using an Al K*α* micro‐focused monochromatized source. The survey, Ga 2p, Ga 3d, Te 3d, and O 1s spectra were got during XPS measurements. All the spectra were calibrated using C 1s peak to 284.8 eV.

The Raman spectra measurements, including the ARPRS characterization, and PL spectra, were conducted using inVia confocal Raman microscope (Renishaw, UK). The wavelength of the exciting laser wavelength is 532 nm, with a spot size less than 1 µm. To minimize the heating effect, the laser power is set as 0.1 mW during Raman measurements. For ARPRS characterization, the GaTe flakes were revolving under the polarization incident laser, while the scattered light with all polarization directions was collected. To analyze the peak position, FWHM, and intensity, the measured Raman peaks were fitted by the Lorentzian/Gaussian function.

For optical reflectivity spectra measurement, a white light beam was used as the excitation source. The reflectivity of the multilayer GaTe before and after exposure to air was measured using an angle‐resolved spectrum system in micro‐region (ARM, Ideaoptics, China).

### Device Fabrication and Measurements

The exfoliated multilayer GaTe flake was transferred on a silicon wafer with 300 nm silicon oxide. Then, GaTe FET was fabricated using a customized silicon shadow mask (Prmat, China) by an e‐beam evaporator (ATTO‐10, Etelux, China). Then, the device was then annealed in an Ar atmosphere at the temperature of 350 ℃ to remove surface oxygen absorption and decrease the contact resistance. As for the GaTe‐O_2_ FET, the electrodes (5 nm Cr/100 nm Au) were defined through the mask‐less UV lithography (DML‐LAB‐1, IOE, China) and then deposited by the e‐beam evaporator (ATTO‐10, Etelux, China). The transport properties were carried out through the Keithley 2636B source meter (Tektronix, USA).

A supercontinuum laser source (SuperK Extreme OCT, NKT Photonics, Denmark) was adopted to provide lasers with different wavelengths and intensities. The current–voltage curves were measured using a Keithley 2636B source meter (Tektronix, USA) with and without laser irradiation. To explore the polarization‐sensitive photo‐response, the laser was passed through the polarizer and half‐wave plate then reached the GaTe photodetector.

### DFT Calculation

The first‐principles calculations were performed using CASTEP modules of Materials studio. The geometry optimizations for GaTe and GaTe—O_2_ were carried out using general gradient approximation applying Perdew–Burke–Ernzerhof (PBE) as correlation functional, with the convergence tolerance of energy 1.0e‐5 eV atom^−1^, maximum force 0.03 eV Å^−1^, maximum stress 0.05 Gpa, and maximum displacement 0.01 Å.

For band structure and DOSs calculations, the Heyd–Scuseria–Ernzerhof functional was adopted, with the pseudopotentials of norm‐conserving, K‐point of 1 × 3 × 1, and relativistic treatment of Koelling‐Harmon. The cutoff energy is set as 590.00 and 800.00 eV for GaTe and GaTe—O_2_, respectively.

### Statistical Analysis

The Raman spectra were baseline subtracted and normalized for comparison, using the software of WiRE 3.4 (Renishaw, UK). The XPS data were analyzed using the software of Avantage (Thermo Scientific, USA). Other data were presented as raw data.

## Conflict of Interest

The authors declare no conflict of interest.

## Supporting information

Supporting Information

## Data Availability

Research data are not shared.
