# Supplementary material for: Breaking the Cut‐Off Wavelength Limit of GaTe through Self‐Driven Oxygen Intercalation in Air
Source: Adv Sci (Weinh). 2021 Dec 30;9(9):2103429. doi: 10.1002/advs.202103429 (PMC8948563; doi:10.1002/advs.202103429)
Supplement: Supplementary file 1 — Supporting Information [file ADVS-9-2103429-s001.pdf]

ADVANCED SCIENCE
Open Access

Supporting Information

for *Adv. Sci.*, DOI 10.1002/advs.202103429

Breaking the Cut-Off Wavelength Limit of GaTe through Self-Driven Oxygen Intercalation in Air

*Renyan Zhang, Yuehua Wei, Yan Kang, Mingbo Pu, Xiong Li, Xiaoliang Ma, Mingfeng Xu and Xiangang Luo**

WILEY-VCH

ADVANCED SCIENCE
Open Access

Supporting Information

for *Adv. Sci.*, DOI: 10.1002/advs.202103429

Breaking the Cut-off Wavelength Limit of GaTe through Self-driven Oxygen Intercalation in Air

Renyan Zhang[#], *Yuehua Wei*[#], *Yan Kang, Mingbo Pu, Xiong Li, Xiaoliang Ma, Mingfeng Xu, Xiangang Luo*[*]

Dr. R. Zhang
State Key Laboratory of Optical Technologies on Nano-Fabrication and Micro-Engineering, Institute of Optics and Electronics, Chinese Academy of Sciences, Chengdu, 610209, China; Division of Frontier Science and Technology, Institute of Optics and Electronics, Chinese Academy of Sciences, Chengdu, 610209, China; College of Advanced Interdisciplinary Studies, National University of Defense Technology, Changsha, 410073, China
Y. Wei
College of Advanced Interdisciplinary Studies, National University of Defense Technology, Changsha, 410073, China
Y. Kang
College of Advanced Interdisciplinary Studies, National University of Defense Technology, Changsha, 410073, China; Beijing Institute for Advanced Study, National University of Defense Technology, Changsha, 410073, China
Prof. M. Pu, Prof. X. Li, Prof. X. Ma, Dr. M. Xu, Prof. X. Luo
State Key Laboratory of Optical Technologies on Nano-Fabrication and Micro-Engineering, Institute of Optics and Electronics, Chinese Academy of Sciences,

Chengdu, 610209, China; Division of Frontier Science and Technology, Institute of Optics and Electronics, Chinese Academy of Sciences, Chengdu, 610209, China

# R.Z. and Y.W. contributed equally to this work.
*Corresponding Author: X.L.: lxg@ioe.ac.cn

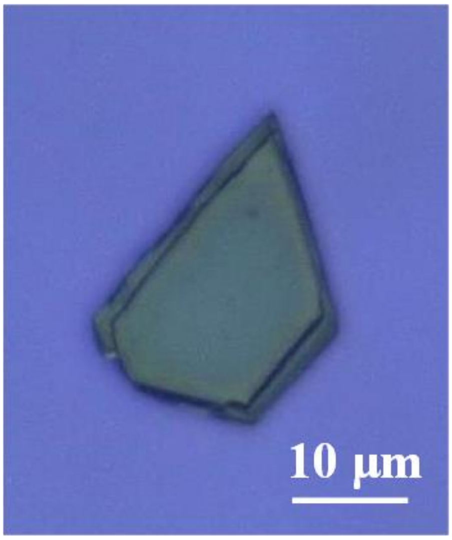

Supplementary Figure S1. Optical image of a typical GaTe flake after exposure to air for 10 days.

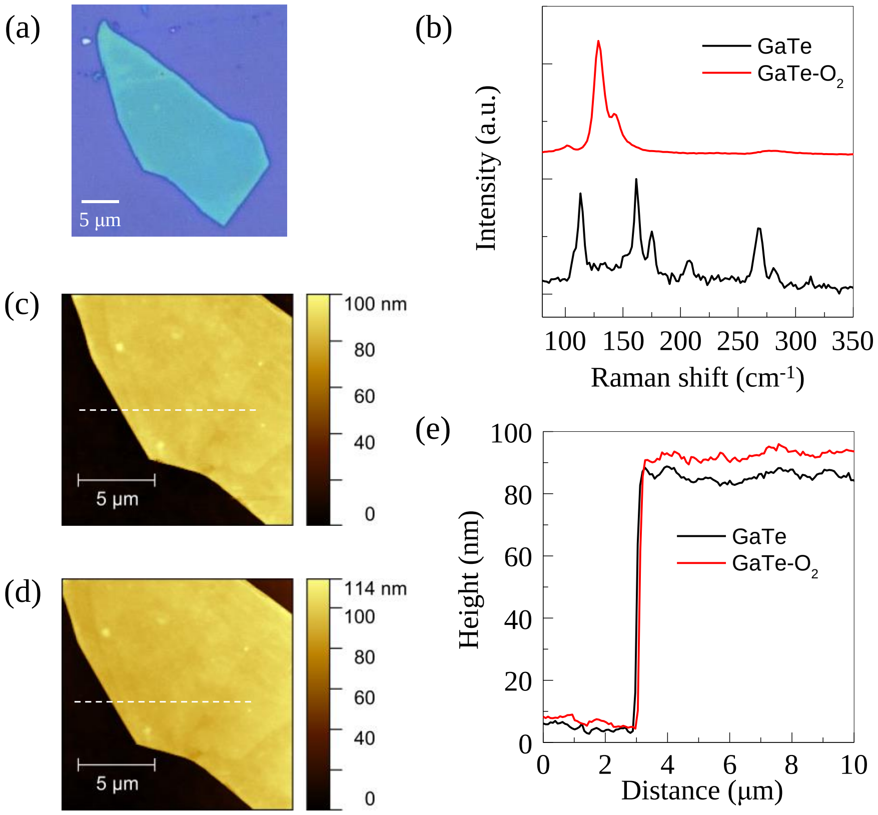

Supplementary Figure S2. (a) Optical image of as exfoliated GaTe flake. (b) The corresponding Raman spectra of annealed GaTe flake (black) and the flake after air exposure for 10 days (red). (c, d) AFM images of GaTe and GaTe-O_2. (e) The corresponding height profile along the line in (c) and (d).

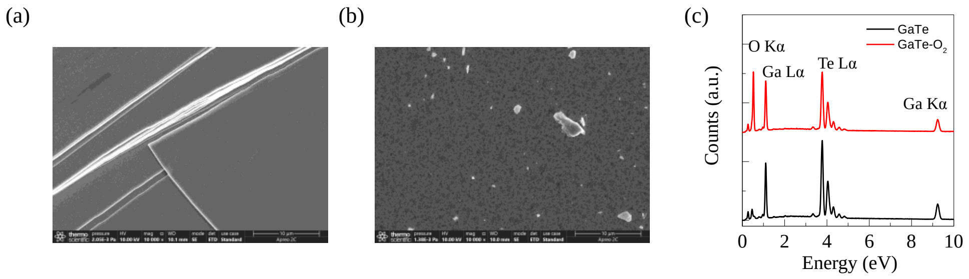

Supplementary Figure S3. (a, b) SEM images of GaTe flakes before and after air exposing. (c) The corresponding EDS spectra.

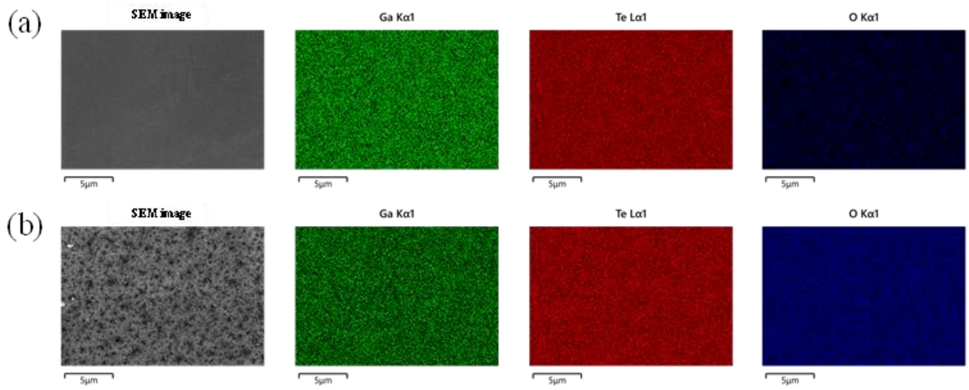

Supplementary Figure S4. (a, b) EDS mapping of GaTe before and after air exposing, including SEM image, Ga element, Te element, and O element, respectively.

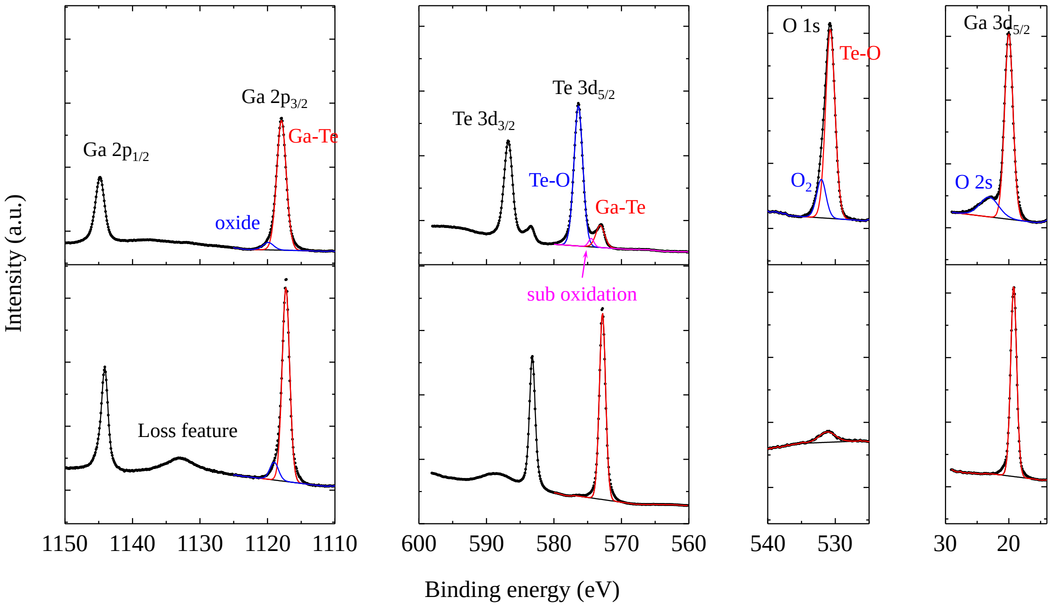

Supplementary Figure S5. XPS spectra for GaTe before (lower panel) and after (upper panel) air exposing, including Ga 2p, Te 3d, O 1s, and Ga3d spectra.

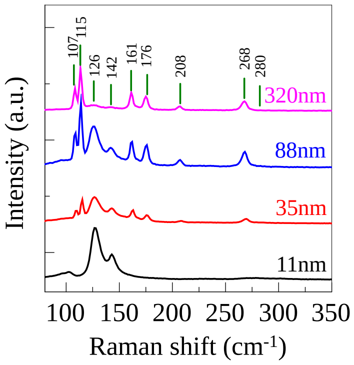

Supplementary Figure S6. Raman spectra of as exfoliated GaTe flakes with different thicknesses. The thickness of GaTe flakes is labeled at the right of the Raman spectra.

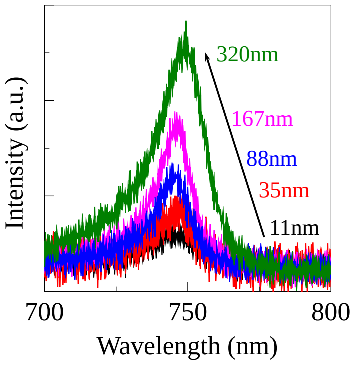

Supplementary Figure S7. PL spectra of as exfoliated GaTe flakes with different thicknesses. The thickness of GaTe flakes is labeled at the right of the PL spectra.

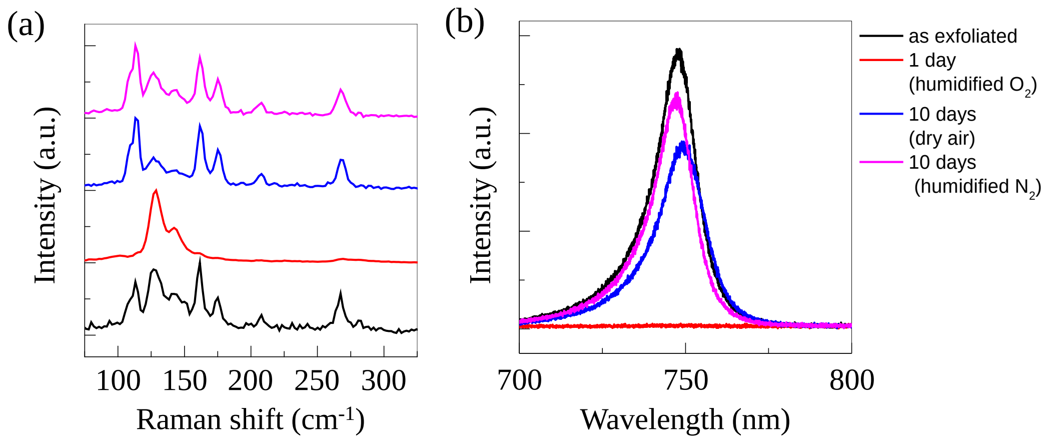

Supplementary Figure S8. Raman (a) and PL (b) spectra for GaTe flakes after being exposed to different atmospheres, including as exfoliated, 1 day in humidified O_2, 10 days in dry air, and 10 days in humidified N_2.

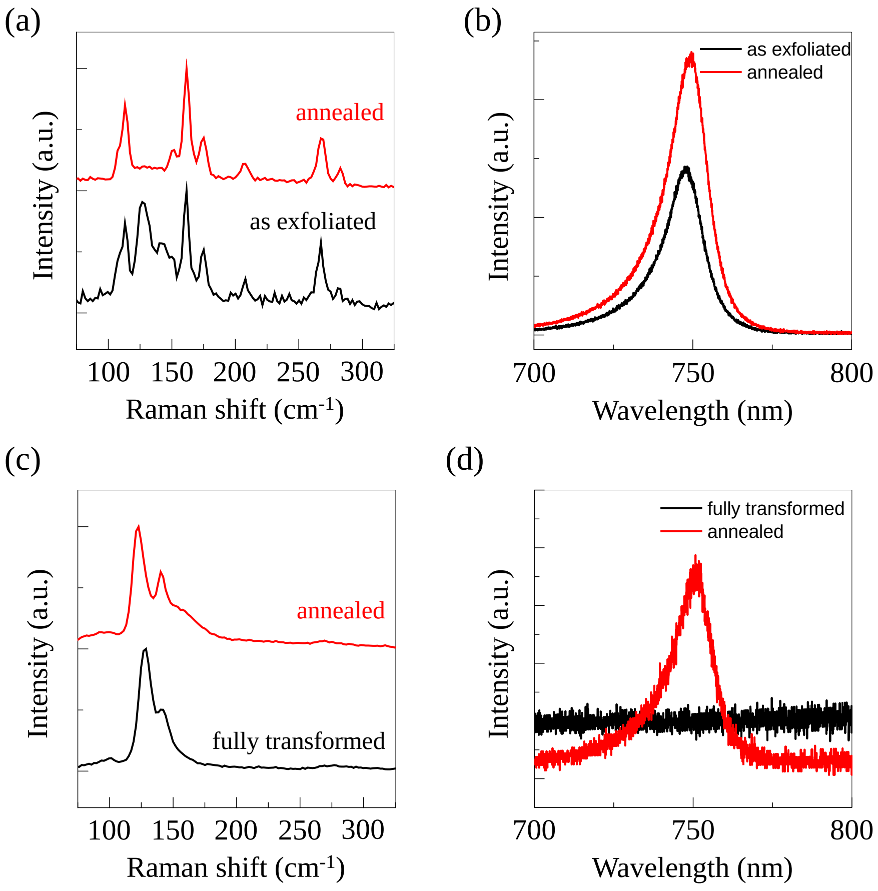

Supplementary Figure S9| (a, b) Raman and PL spectra for as exfoliated GaTe before and after annealing in Ar at the temperature of 350 ℃ for 30 minutes, respectively. (c, d) Raman and PL spectra for fully transformed GaTe-O_2 before and after annealing in Ar at the temperature of 350 ℃ for 30 minutes, respectively.

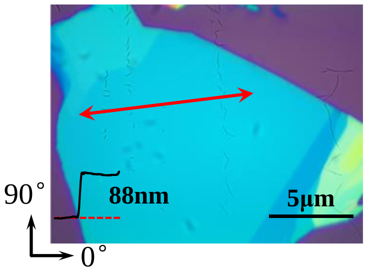

Supplementary Figure S10| The optical image of a typical as exfoliated GaTe flakes. The y crystal direction is indicated by the red line. Inset: The corresponding AFM high profile of GaTe flakes. The 0° and 90° orientations correspond to the horizontal and vertical direction, respectively.

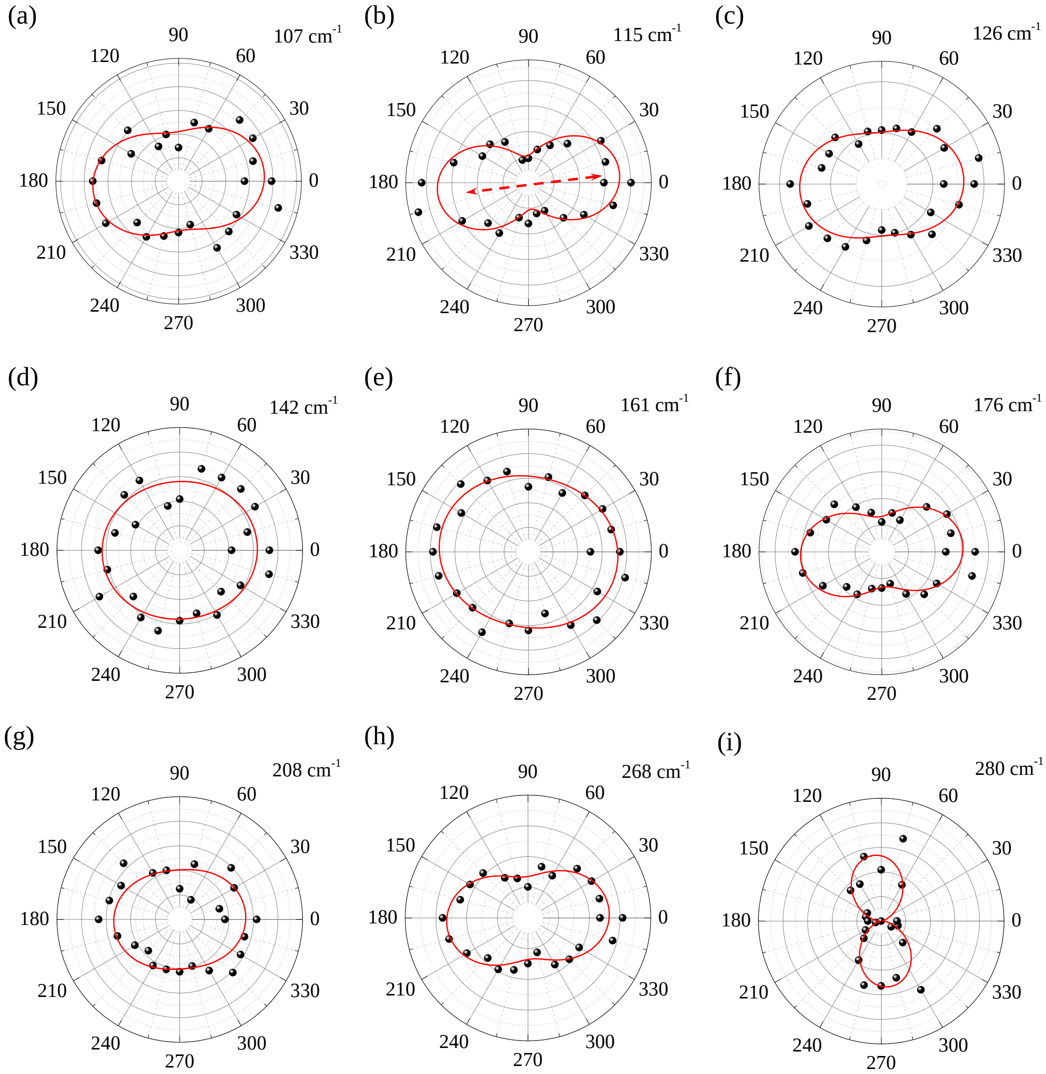

Supplementary Figure S11| Corresponding polar plots of the measured and fitted peak intensities for the as exfoliated GaTe flakes in Figure S10. (a, b, c, d, e, f, g, h, and i) The Raman intensity vs polarization angle for the Raman mode of 107, 115, 126, 142, 161, 176,

208, 268, and 280 cm^{-1}, respectively. The black dots and red curves are the experimental data and fitted curves, respectively.

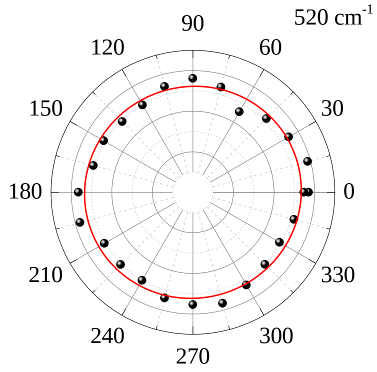

Supplementary Figure S12| The Raman intensity vs polarization angle for the Raman mode of silicon (520 cm^{-1}).

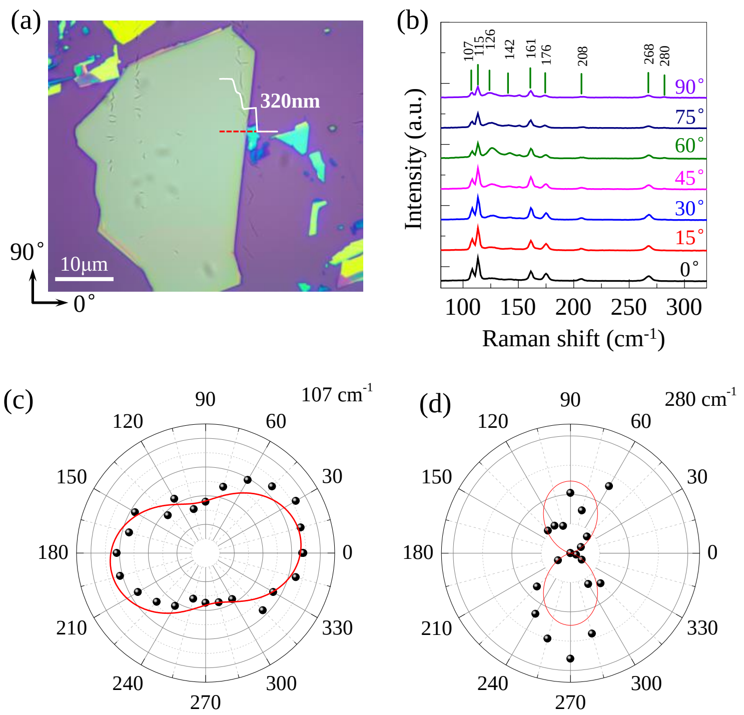

Supplementary Figure S13| Raman characterization for the as exfoliated GaTe flakes with a thickness of ~ 320 nm. (a) Optical image of the 320 nm thick GaTe flakes. Inset: The corresponding AFM high profile of GaTe flakes. The 0° and 90° orientations correspond to the horizontal and vertical direction, respectively. (b) Angle-resolved polarized Raman spectra of GaTe in (a), with the sample rotated from 0 to 360° with the laser polarization direction. The excitation laser wavelength is 532 nm. The Raman modes are marked. Zero

degrees is defined as the horizontal direction of the flakes. (c, d) Corresponding polar plots of the measured and fitted peak intensities of the two typical Raman modes with Raman shift valve labeled at the top right corner of each panel: 107 cm^{-1} and 280 cm^{-1}, respectively. The black dots and red curves are the experimental data and fitted curves, respectively.

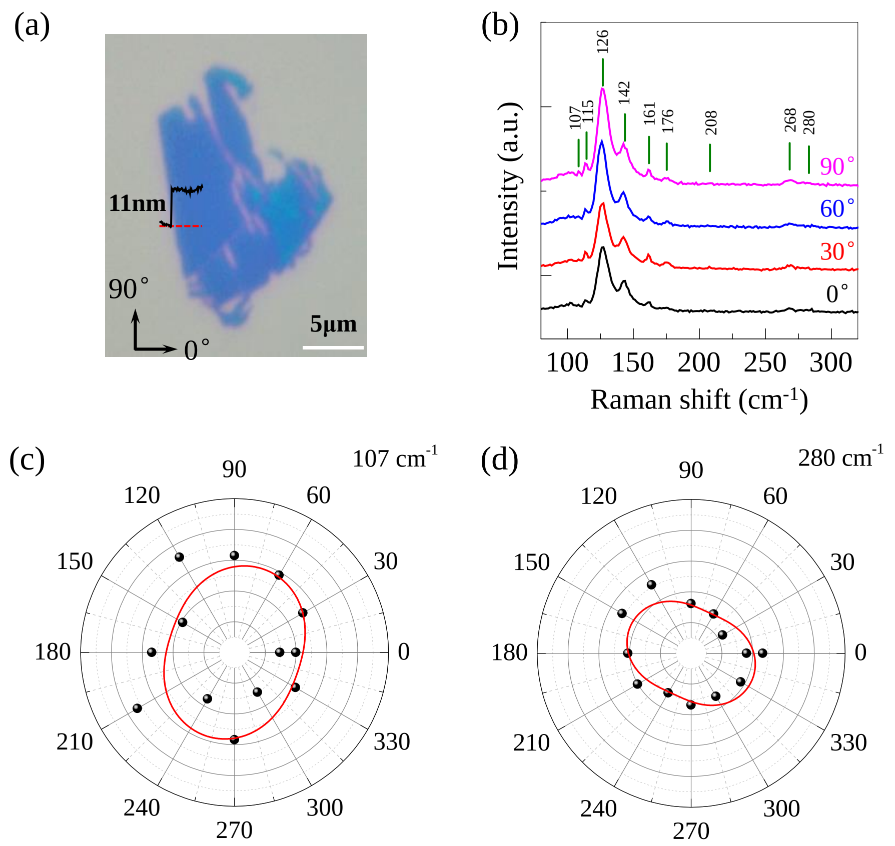

Supplementary Figure S14| Raman characterization for the as exfoliated GaTe flakes with a thickness of ~ 11 nm. (a) Optical image of the 11 nm thick GaTe flakes. Inset: The corresponding AFM high profile of GaTe flakes. The 0° and 90° orientations correspond to the horizontal and vertical direction, respectively. (b) Angle-resolved polarized Raman spectra of GaTe in (a), with the sample rotated from 0 to 360° with the laser polarization direction. The excitation laser wavelength is 532 nm. The Raman modes are marked. Zero degrees is defined as the horizontal direction of the flakes. (c, d) Corresponding polar plots of the measured and fitted peak intensities of the two typical Raman modes with Raman shift valve labeled at the top right corner of each panel: 107 cm^{-1} and 280 cm^{-1}, respectively. The black dots and red curves are the experimental data and fitted curves, respectively.

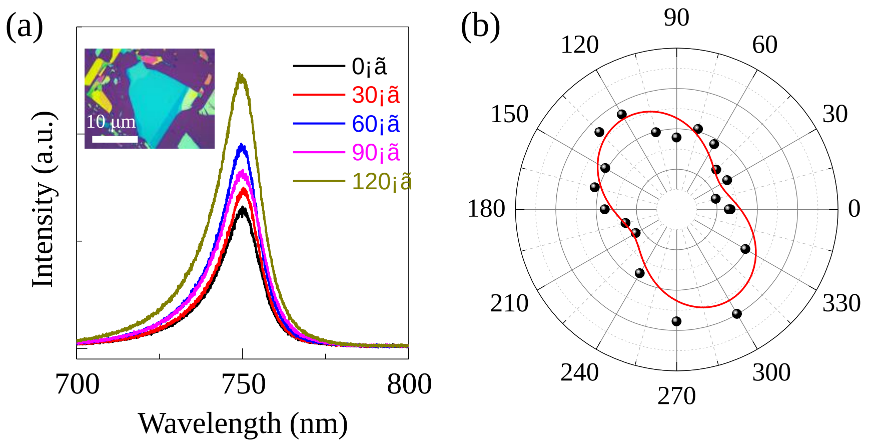

Supplementary Figure S15| Polarization-sensitive PL spectra of the as exfoliated GaTe flakes. (a) Angle-resolved polarized PL spectra of GaTe. Inset: corresponding optical images of the GaTe flake. The excitation laser wavelength is 532 nm. (b) Corresponding polar plots of the measured and fitted peak intensities of the PL spectra.

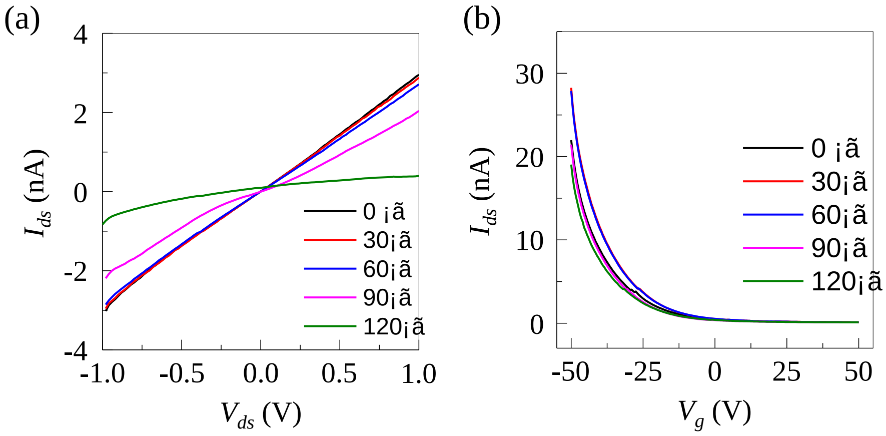

Supplementary Figure S16| (a, b) Direction-dependent I_{ds}-V_{ds} curves and transfer curves for devices in Figure 1f, respectively.

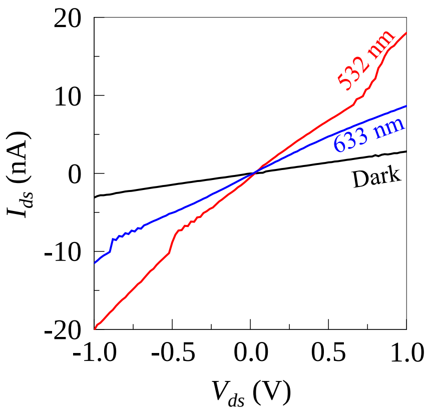

Supplementary Figure S17| I_{ds} - V_{ds} curves of the GaTe device along 0°-180° direction in Figure 1f with and without laser radiation (50 mW/cm^2).

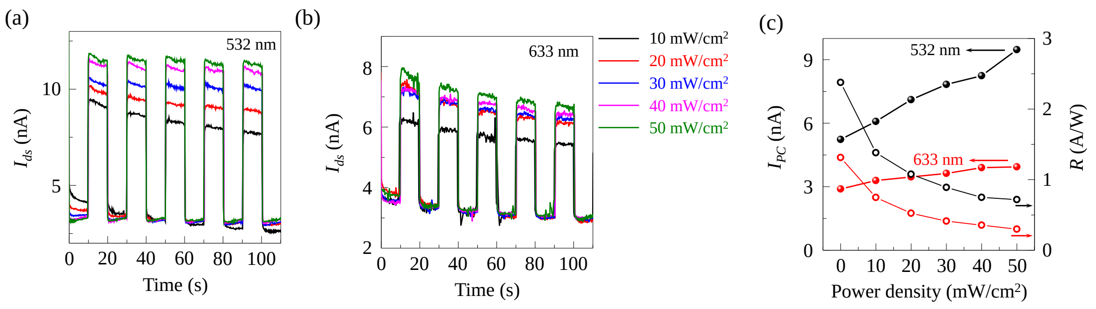

Supplementary Figure S18| (a, b) The dependence of I_{ds} with times with different laser power densities for the wavelength of 532 nm and 633 nm, respectively. (c) Photocurrent and photoresponsivity of GaTe device (Figure 1f) as a function of laser power density for the wavelength of 532 nm and 633 nm.

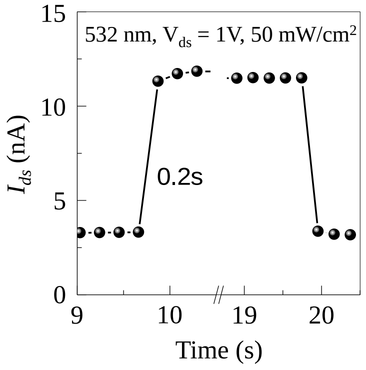

Supplementary Figure S19| The response time for GaTe photodetector (Figure 1f) along 0°-180° direction in Figure 1f.

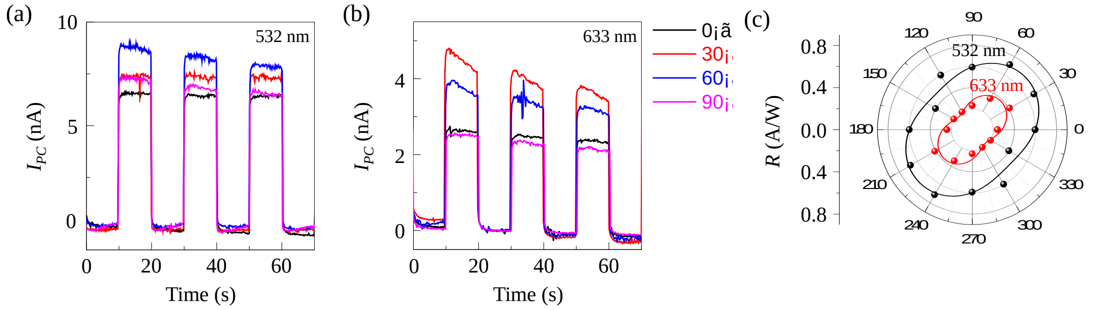

Supplementary Figure S20| (a, b) The dependence of I_{ds} with times with different electrode pairs (Figure 1f) for the wavelength of 532 nm and 633 nm, respectively. (c) Direction-dependent photo-responsivity for the wavelength of 532 nm and 633 nm.

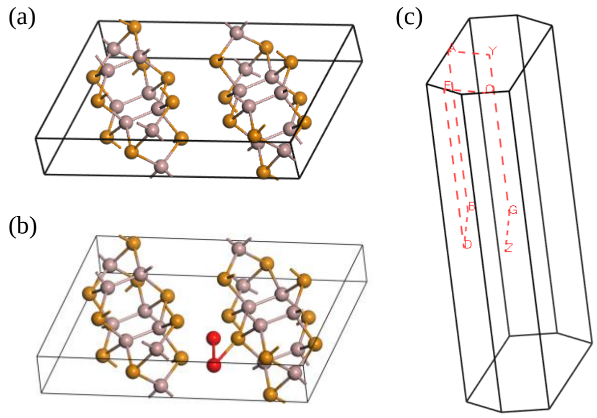

Supplementary Figure S21| (a, b) Unit cell for GaTe and GaTe-O_2, respectively. (c) The first Brillouin zone of GaTe.

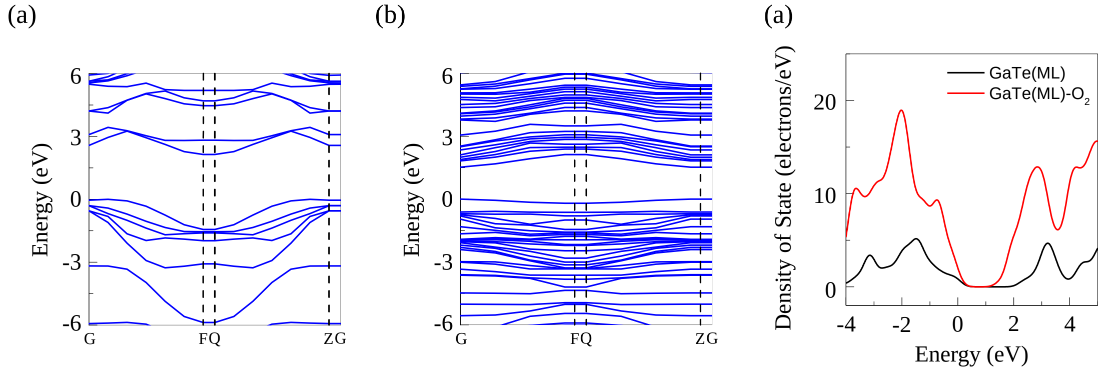

Supplementary Figure S22| (a, b) Calculated electronic band structures of monolayer GaTe and GaTe-O_2, respectively. (c) The calculated density of states (DOS) of monolayer GaTe and GaTe-O_2.

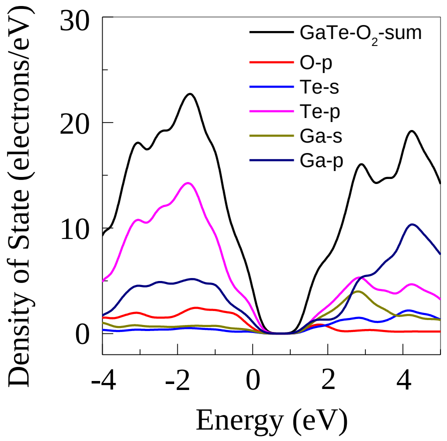

Supplementary Figure S23| Orbital-projected density of states of GaTe-O_2 near the bandgap.

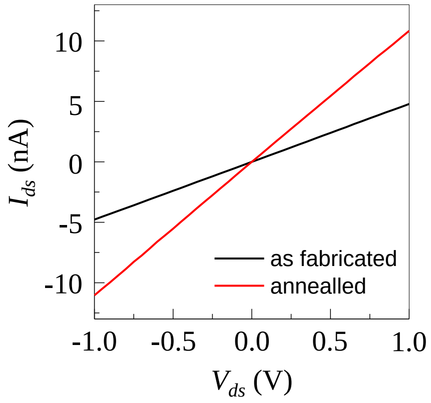

Supplementary Figure S24| I_{ds} - V_{ds} curves for GaTe device in the inset of Figure 3a in dark before and after annealing in Ar at the temperature of 350 °C for 30 minutes.

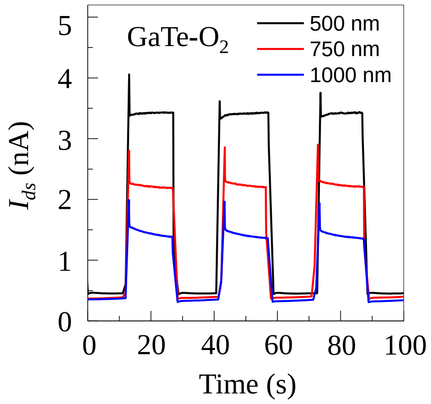

Supplementary Figure S25| The dependence of I_{ds} with times with different wavelength for GaTe-O_2 device in Figure 3b inset.

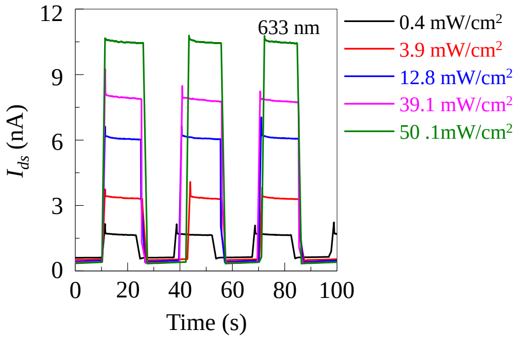

Supplementary Figure S26| The dependence of I_{ds} with times with different laser powder density for the wavelength of 633 nm for the device in Figure 3b inset, respectively.

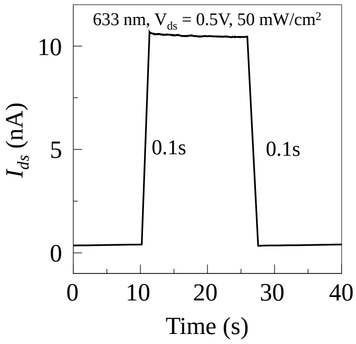

Supplementary Figure S27| The response time for GaTe-O_2 photodetector in Figure 3b inset.

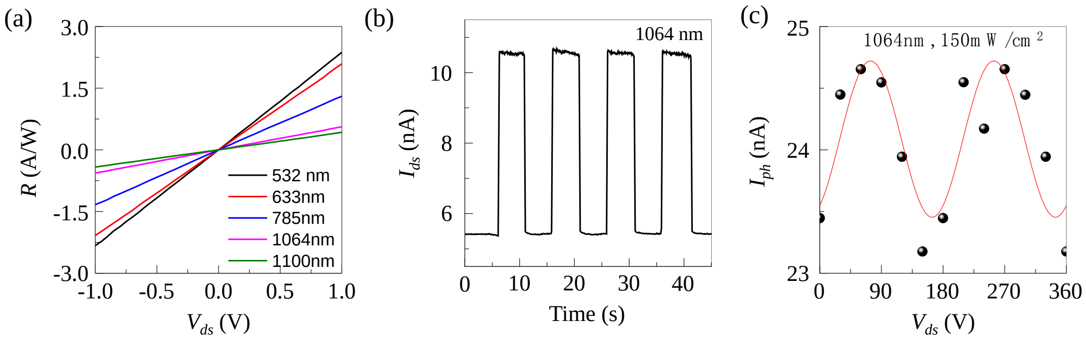

Supplementary Figure S28| (a) R- V_{ds} curves for GaTe-O_2 device in Figure 3b inset after exposure to air for 2 weeks under laser radiation (10 mW/cm^2) with different wavelengths. (b) The dependence of I_{ds} with times for GaTe-O_2 device in Figure 3b inset with laser radiation (1064 nm, 130 mW/cm^2). (c) Angle-resolved photocurrent of GaTe-O_2 photodetector (Figure 3b inset) under 1064 nm laser radiation with the power density of 150 mW/cm^2.

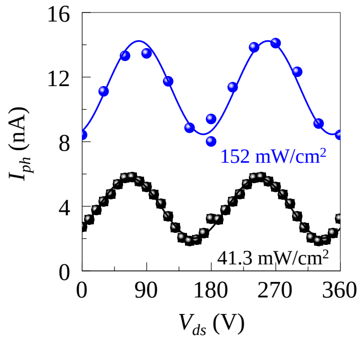

Supplementary Figure S29| Angle-resolved photocurrent of GaTe photodetector (Figure 3b inset) under 1100 nm laser radiation with different power densities.

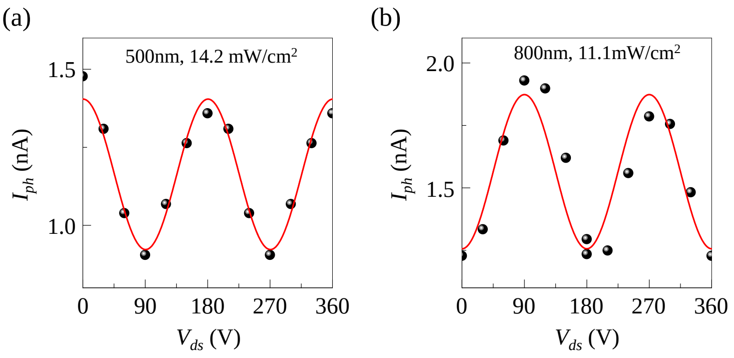

Supplementary Figure S30| Angle-resolved photocurrent of GaTe photodetector (Figure 3b inset) under 500 nm and 800 nm laser radiation.
